# Electroactive Biofilms of Activated Sludge Microorganisms on a Nanostructured Surface as the Basis for a Highly Sensitive Biochemical Oxygen Demand Biosensor

**DOI:** 10.3390/s22166049

**Published:** 2022-08-12

**Authors:** Saniyat Kurbanalieva, Vyacheslav Arlyapov, Anna Kharkova, Roman Perchikov, Olga Kamanina, Pavel Melnikov, Nadezhda Popova, Andrey Machulin, Sergey Tarasov, Evgeniya Saverina, Anatoly Vereshchagin, Anatoly Reshetilov

**Affiliations:** 1 Laboratory of Biologically Active Compounds and Biocomposites, Tula State University, Lenin Pr. 92, Tula 300012, Russia; 2 M. V. Lomonosov Institute of Fine Chemical Technologies, MIREA—Russian Technological University, Prosp. Vernadskogo 86, Moscow 119571, Russia; 3 Federal State Budgetary Institution of Science Institute of Physical Chemistry and Electrochemistry of the Russian Academy of Sciences, Leninsky Prosp., 31 k. 4., Moscow 119071, Russia; 4 Institute of Biochemistry and Physiology of Microorganisms of the Russian Academy of Sciences—A Separate Subdivision of the FRC Pushchino Scientific Center for Biological Research of the Russian Academy of Sciences, Prosp. Science 3, Pushchino 142290, Russia; 5 N. D. Zelinsky Institute of Organic Chemistry, Leninsky Pr. 47, Moscow 119991, Russia

**Keywords:** electroactive biofilms, bioelectrocatalysis, activated sludge, carbon nanotubes, BOD biosensors, nanostructured surface, electron transport mediator, redox-active polymers

## Abstract

The possibility of the developing a biochemical oxygen demand (BOD) biosensor based on electroactive biofilms of activated sludge grown on the surface of a graphite-paste electrode modified with carbon nanotubes was studied. A complex of microscopic methods controlled biofilm formation: optical microscopy with phase contrast, scanning electron microscopy, and laser confocal microscopy. The features of charge transfer in the obtained electroactive biofilms were studied using the methods of cyclic voltammetry and electrochemical impedance spectroscopy. The rate constant of the interaction of microorganisms with the extracellular electron carrier (0.79 ± 0.03 dm^3^(g s)^−1^) and the heterogeneous rate constant of electron transfer (0.34 ± 0.02 cm s^−1^) were determined using the cyclic voltammetry method. These results revealed that the modification of the carbon nanotubes’ (CNT) electrode surface makes it possible to create electroactive biofilms. An analysis of the metrological and analytical characteristics of the created biosensors showed that the lower limit of the biosensor based on an electroactive biofilm of activated sludge is 0.41 mgO_2_/dm^3^, which makes it possible to analyze almost any water sample. Analysis of 12 surface water samples showed a high correlation (R^2^ = 0.99) with the results of the standard method for determining biochemical oxygen demand.

## 1. Introduction

Monitoring of the quality of wastewater treatment and the state of natural waters experiencing a high anthropogenic load from industrial enterprises is a priority task to prevent environmentally hazardous situations. Integrated analytical systems allow tracking the spread of pollution, as well as quickly and accurately determining the water quality in a particular region. The last decade has been marked by the intensive use of microorganisms for the quantitative determination of integral indicators such as biochemical oxygen demand (BOD) [1,2]. BOD is one of the most widely used indicators for monitoring the purity of aquatic environments and represents, by definition, the amount of oxygen required for the biochemical oxidation of organic substances contained in a water sample. The accepted method for determining BOD by the international standard ISO 5815-1:2003 [3]. The main disadvantage of the technique is the duration of the analysis (at least 5 days for BOD_5_), which leads to the development of express methods. Biosensor analyzers which are based on the use of microorganisms that metabolize a wide range of organic compounds, which make it possible to determine the BOD index in a few minutes, have been created [4].

As a rule, biosensors make it possible to determine the BOD index in the range of 2–300 mgO_2_/dm^3^ in a few minutes. At the same time, the correlation coefficient between the BOD values determined using a biosensor and the standard method ranges from 0.6 to 0.98 [4,5,6]. However, a large number of publications regularly published on this topic indicate that characteristics have not yet been obtained that would stop the process of further search for improvements for this analysis. The most urgent problem for the development of the BOD sensors is to increase the sensitivity of the analysis, since most of the described biosensors do not allow determining BOD in clean surface waters, in which BOD is less than 2 mgO_2_/dm^3^ [6,7].

The most commercially available biosensors for environmental monitoring are based on the electrochemical determination of the rate of the reaction product formation of microorganisms or their consumption of oxygen. Modern approaches for the detection of organic compounds comprise the use of biosensors based on the using of mediators of electron transport or direct electron transfer [8]. Such approaches provide high sensitivity of the method, a short analysis time, and the possibility of significant miniaturization of microbial biosensor systems, which can be traced as a trend in the modern glucometers.

As a rule, a suspension of one [9] or several [10] microorganisms, or activated sludge [11] are used to form BOD biosensors. Currently, biofilms play a special role in bioelectrochemical catalysis [12]. For example, one study [13] presented a BOD biosensor based on a biofuel cell and a biofilm of *Geobacter* sp. bacteria, which was grown in a medium with ethanol as the sole carbon source. The range of determined BOD_5_ values was 174–1200 mgO_2_/dm^3^, and the analysis time was 17.5 h. Therefore, the developed system could not be used for express analysis of surface waters with low BOD values. A biosensor based on an electroactive biofilm of bacteria *Shewanella loihica* made it possible to determine BOD_5_ in the range of 0–435 mgO_2_/dm^3^ in less than 1 h, but had an analysis error of about 20% [14], which is not acceptable for an accurate assessment of the degree of wastewater pollution. In another paper [15], an approach was proposed to obtain a biofilm of a community of microorganisms from wastewater treatment plants for the formation of a BOD biosensor. The range of determined BOD_5_ values was 2–64 mgO_2_/dm^3^, the measurement time was 10 min, and the long-term stability was 2 months.

Electroactive microorganisms can form a stable biofilm on the electrode surface due to the formation of an extracellular polymeric substance, due to which the microorganisms are attached to the electrode surface. Two mechanisms of charge accumulation and transfer were proposed after studying the processes of the formation of electroactive biofilms. The first mechanism occurs with the help of biopolymers such as polyhydroxyalkanoates [16]. The second mechanism occurs with the help of reduced cellular components, such as cytochromes [17], located on the cell surface or flavins [18], which are either bound to proteins on the cell wall or diffuse freely in the biofilm. It is currently unknown to what extent these electron accumulation mechanisms contribute to the overall generation of the analytical signal [19]. A deeper understanding of the processes in electroactive biofilms is required to complement the current knowledge about the functioning of biofilms. It is necessary to carry out an electrochemical study of the mechanisms of the extracellular electron transfer in biofilms (cyclic voltametry, electrochemical impedance spectroscopy), as well as in situ microscopic measurements (scanning electron microscopy, confocal laser microscopy) [20,21]. As a result, the structure of the biofilm will be determined depending on the mechanism of its formation on the electrode.

Often, biofilms have limited bioelectrocatalytic activity [22], which is probably due to the low content of redox-active compounds in bionanowires or due to the existence of a kinetic hindrance, due to which it becomes impossible to transfer electrons through bionanowires. In addition, due to the weak attachment of the biofilm to the surface of the conventional electrodes (which are most often based on graphite), the efficiency of electron transport is significantly reduced due to the kinetic limitation of the surface transfer of electrons to the electrode [23]. Nanomaterials can increase the surface area for biofilm growth, enhance cell attachment to transducers, and increase system conductivity [24,25]. The introduction of nanomaterials into the structures of biopolymers makes it possible to improve the analytical characteristics of biosensors. In the field of biosensor analysis, the use of nanotubes made it possible to achieve direct transfer from the active center of enzymes to the electrode [26,27].

Therefore, in the last few years there has been a significant increase in the number of publications in which the successful integration of biofilms and nanomaterials has been noted, but these works are mainly related to the development of biofuel cells and are often of a purely applied nature. The purpose of this work was to comprehensively study the electrochemical parameters of the electron transfer and biological and physical properties of biofilms, which will allow the development of a biosensor device for the early warning of the natural water pollution.

Biofilms have well-known properties, including stability over time and a high resistance to factors which are negative for microorganisms. It is important to note that these properties will be used to create sensitive and stable sensor systems for creation a BOD biosensor. The use of microorganisms capable of forming electroactive biofilms will lead to the abandonment of the use of exogenous electron transport mediators, which will greatly simplify and reduce the cost of the analytical systems.

## 2. Materials and Methods

### 2.1. Reagents and Materials

Ferrocene (Dia-m, Moscow, Russia) was used as an electron transport mediator. Nutrient medium was prepared using D-glucose (Panreac, Barcelona, Spain), peptone (Conda, Madrid, Spain), tryptone (Conda, Madrid, Spain), and yeast extract (Helicon, Moscow, Russia). Graphite powder with a particle size of 75 μm with a high frequency of 99.997% (Fluka, Darmstadt, Germany), paraffin oil (Fluka, Germany), carbon nanotubes of the “Taunit” («NanoTechCenter», Tambov, Russia) series with an outer diameter of 10–30 nm, length ≥ 2 μm, and amount of impurities ≤ 1%, and a dialysis membrane with a transmission limit of 14 kDa (Roth, Germany) were taken to create a working graphite paste electrode. Biosensor measurements were performed using a sodium-potassium phosphate buffer solution pH = 6.8 (33 mM KH_2_PO_4_ + 33 mM Na_2_HPO_4_, Dia-m, Moscow, Russia).

### 2.2. Cultivation of Microorganism Cells

Activated sludge was taken from the wastewater treatment plant for the production of chemical fertilizers. Cells of activated sludge microorganisms were grown on a liquid Lysogeny broth (LB) medium with composition: tryptone—10 g/dm^3^, yeast extract—5 g/dm^3^, and sodium chloride—10 g/dm^3^. The medium for growing cells was sterilized by autoclaving at a pressure of 1.15 atm. within 45 min. Cells were grown aerobically for 20–24 h in shaking flasks with a volume of 750 cm^3^ at a temperature of 29 °C. Then the grown biomass was centrifuged at room temperature in an Eppendorf centrifuge (Germany) at 10,000 rpm for 10 min and washed from the culture medium with 20 mM phosphate buffer pH = 6.8 (2 times). The settled cells were washed in a fresh portion of the buffer, divided into portions, and besieged in a centrifuge for 10 min at 10,000 rpm. The washed biomass was weighed and stored in microtubes at −10 °C.

### 2.3. Formation of a Working Mediator Electrode

A working graphite-paste electrode (GPE) was formed by filling a plastic tube with a working surface area of 6.3 mm^2^ with the prepared graphite powder-mineral oil paste. The electrode surface was mechanically cleaned and polished. A weighed portion of ferrocene (10 mg) was dissolved in 0.5 cm^3^ of acetone and added to 90 mg of graphite powder (the mass fraction of ferrocene from the total mass of the powder was 10% wt.%) to modify the working electrode using an exogenous electron transport mediator. Then, 0.1 cm^3^ of deionized water was added to 5 mg of multiwalled carbon nanotubes (diameter 10–30 nm, specific surface ≥ 270 m^2^/g, NanoTechCenter, Moscow, Russia), and then left in an ultrasonic bath for 15 min to obtain a modification of the working electrode with CNT (carbon nanotubes). Afterwards, 0.01 cm^3^ of a CNT suspension was applied to a cleaned graphite-paste electrode and left to dry.

Microorganisms were applied to the surface of the modified working electrode in the form of a suspension with a titer of 330 mg/cm^3^ in an amount of 0.01 cm^3^ to form electrodes with a cell suspension. Therefore, the specific mass of microorganisms on the surface of the graphite-paste electrode was 0.52 mg/mm^2^. A dialysis membrane was used to fix the suspension of microorganisms on the electrode surface. The modified working electrode was placed in sterile test tubes with LB nutrient medium (15 cm^3^) and microorganisms to form electrodes with cell biofilms. The experiment was carried out at a temperature of 29 °C for 53 h. 

### 2.4. Biosensor Measurements

Electrochemical measurements were performed using an IPC-micro galvanopotentiostat (NTF Volta, St. Petersburg, Russia) integrated with a personal computer. The range of possible recorded currents was from 5 to 20 μA. The potential measurement error did not exceed 0.1 mV for an interval of ±5 mV. The operating potential was +280 mV for electrodes based on ferrocene and +250 mV for electrodes based on electroactive biofilms. The measurements were carried out at a temperature of 20 °C; the volume of the measuring cell was 5 cm^3^. The measurements were carried out with continuous stirring of the solution using a magnetic stirrer (300 rpm). After a stable current level was established, the amount of the test compound required to obtain a given concentration was introduced into the cells. The cells were washed with potassium-sodium-phosphate buffer solution pH = 6.8 after each measurement. The measured parameter of the sensor signal was the amplitude of the change in the current strength, which was defined as the difference between the final and initial values of the currents before and after the introduction of the test compound into the measuring cuvette.

### 2.5. Voltammetric Measurements

Voltammograms (CVs) were recorded using a three-electrode scheme using an Ecotest-VA voltammetric analyzer (Econix-Expert, Moscow, Russia). A modified graphite-paste electrode was used as a working electrode, and a platinum electrode was used as a counter electrode. A saturated silver chloride electrode (Ag/AgCl) was used as a reference electrode. Cyclic voltammograms were recorded at a sweep rate of 20–100 mV/s in potassium-sodium-phosphate buffer (pH = 6.8).

### 2.6. Electrochemical Impedance Spectroscopy (EIS)

The impedance characteristics were measured on a VersaSTAT 4 galvanopotentiostat (Ametek Inc., Berwyn, PA, USA). A three-electrode measurement scheme was used to obtain spectra, where the working electrode was a graphite-paste electrode, and a platinum plate with an area of 30 mm^2^ served as a counter electrode. A silver chloride electrode was used as a reference electrode. Impedance measurements were carried out at an applied potential of +350 mV (vs. Ag/AgCl) in the frequency range from 40 kHz to 0.02 Hz and a voltage modulation amplitude of 10 mV. The measurements were carried out in 25 mM potassium phosphate buffer solution (pH = 6.8) containing 10 mM sodium chloride.

### 2.7. Scanning Electron Microscopy (SEM)

The electron microscopic analysis of the samples was carried out using a JSM-6510 LV scanning electron microscope (JEOL, Tokyo, Japan) in a low vacuum (30 Pa) mode during registration of secondary electrons.

### 2.8. Optical Microscopy

Biofilm formation was studied using a Nikon Eclipse Ci optical light microscope (Nikon, Tokyo, Japan) equipped with a ProgRes SpeedXT core5 camera (Jenoptik, Jena, Germany). The observation was carried out in the phase contrast mode.

### 2.9. Laser Confocal Scanning Microscopy (LKSM)

A visualization of biofilms was carried out by laser confocal scanning microscopy (microscope Leica SP5 (Leica, Wetzlar, Germany)). The polysaccharide matrix was stained with the ConA lectin conjugated with the fluorescent dye Alexa Fluor 488 (W11261 ThermoFisher, Waltham, MA, USA) [28]. Cells were visualized using SYTO^®^ 11 fluorescent dye (S7573 ThermoFisher, USA) diluted 1:1000 in phosphate buffer. Images were acquired with an argon laser at 488 nm (for detection of the fluorescent dye Alexa Fluor 488) and 594 nm (for detection of SYTO^®^ 11) using the Nomarski contrast method to detect unstained particles. The resulting images were analyzed using the ImageJ software package with the BioFormats 5.8.2 plugin.

### 2.10. Control of Biofilm Development

The physiological activity of microorganisms in biofilms was recorded using a breath test based on the ability of cellular oxidoreductase enzymes to reduce the tetrazolium dye 3-(4,5-dimethylthiazol-2-yl)-2,5-diphenyl-tetrazolium bromide (MTT) into insoluble formazan (MTT-test) [29]. Microorganisms were grown in sterile Eppendorf microtubes at 29 °C. Biofilm formation was determined after removal of the growth medium. A 0.1% solution of MTT with a volume of 0.5 mL was added to the microtubes to control the formation of biofilms, and then they were incubated for 1 h at 29 °C. After staining with tetrazolium dye, the liquid was drained and washed with water, and 96% ethanol was added and left for 45 min to extract the dye from biofilms. The optical density of the extract was determined on an Expert-003 photometer (Econix-Expert, Russia) at a wavelength of 590 nm. The levels of biofilm formation were judged from the color intensities of the obtained solutions.

### 2.11. Determination of the Number of Viable Cells of Microorganisms by the Koch Method

A number of successive dilutions of suspensions of microbial cells were prepared (the degree of dilution was 10^−6^). A sowing of microorganism suspensions was carried out by the surface method on a solid nutrient medium from the last three dilutions (4 parallel inoculations were carried out). Colonies were counted after 7 days of incubation.

### 2.12. Determination of BOD by the Standard Dilution Method

The dilution method was used as a reference method for determining BOD_5_. The analysis was carried out in accordance with ISO: Water quality [3]. Dissolved oxygen content was determined using an EXPERT-001-4.0.1 BOD thermooximeter (Econix-Expert, Russia). 

## 3. Results

### 3.1. Formation of Microorganisms’ Biofilms on the Electrode Surface

The use of an immobilized cell suspension is a classic approach to the creation of microbial biosensors. At the same time, an approach based on growing electroactive biofilms with desired properties on the surface of sensors made of conductive graphite materials is innovative. [5]. Currently, there are many attempts to integrate electroactive biofilms into microbial fuel cells [24], but the possibilities of their use in biofilms are much wider. In this work, an activated sludge bacteria was used to create a bioreceptor element of the BOD biosensor, since it is used for the standard method of BOD analysis [3]. This led to a high correlation of biosensor data with the results of the classical method [4]. In addition, activated sludge and microorganisms isolated from it were successfully used to create BOD biosensors [11,30,31].

The MTT breath test was used to monitor the physiological activity of microorganisms in biofilms on the surface of sensitive elements in an in vitro experiment. The formation of biofilms was recorded by an increasing the intensity of microorganisms staining in purple. Based on the results, the dependence of the optical density on the biofilm growth time was plotted. The increase in optical density indicates the process of increasing the physiological activity of the biomass, due to the reproduction of microorganisms, the formation, and growth of a biofilm. The time of maximum physiological activity for activated sludge microorganisms was 53 h, after which it gradually decreased. The formation of a biofilm during the period of maximum physiological activity of cells was confirmed by optical microscopy with phase contrast.

**Figure 1:**
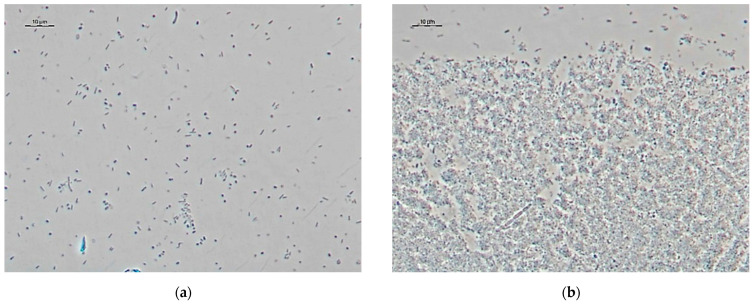

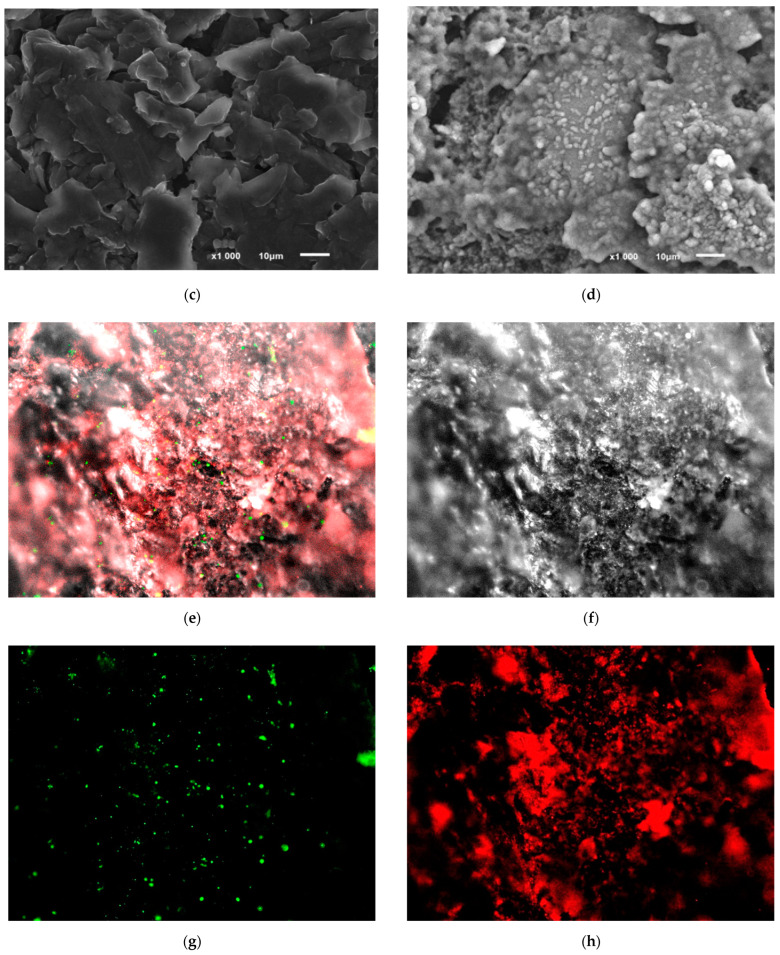
Microscopic studies of the formation of biofilms of activated sludge: (**a**) photograph of optical microscopy for suspensions of activated sludge microorganisms; (**b**) photographs of optical microscopy for activated sludge biofilm after 53 h of growth; (**c**) surface of pure GPE obtained by SEM; (**d**) biofilm of activated sludge on the surface of the GPE, obtained by SEM; (**e**) integrated image of the electrode surface obtained by the LKSM method; (**f**) image of the electrode surface in the Nomarsky contrast mode, obtained by the LKSM method; (**g**) is the image of the electrode surface in the conA dye display mode, obtained by the LKSM method; (**h**) image of the electrode surface in the display mode of the SYTO 11 dye, obtained by the LKSM method.

As can be seen from Figure 1b, the activated sludge biofilm after 53 h of growth is a population of various microorganisms that differ in morphology. During the formation of a biofilm, the formation of a polysaccharide matrix is clearly visible, in contrast to a suspension; in addition, a clear boundary between the formation of a biofilm and free-living microorganisms is visible.

The formation of biofilms of activated sludge microorganisms on the working graphite-paste electrode surface was shown using the method of scanning electron microscopy (Figure 1c,d). The used graphite-paste electrode has a developed surface, which makes it possible to reliably hold the biomaterial on the surface (Figure 1c). The obtained images clearly show the porous structure of the biofilm (Figure 1d). The formed biofilms rather densely cover the electrode surface and form a continuous matrix.

Biofilm growth on the surface of graphite materials can be characterized by the formation of an extracellular polysaccharide matrix, which facilitates cell adhesion to the surface. The method of laser confocal microscopy (Figure 1e–h) was used to study this process.

In Figure 1e, polysaccharides are colored red, and nucleic acids are colored green. The presence of nucleic acids indicates the uniform distribution of cells on the graphite surface (Figure 1g). Bacteria attached to the electrode surface and formed a biofilm. This is evidenced by the intense staining of extracellular polysaccharides (Figure 1h), which occurs due to the binding of the ConA dye to terminal mannosyl and terminal glucosyl residues in polymeric compounds secreted by microorganisms in the biofilm [32]. Thus, according to the results of complex microscopic studies, it can be assumed that homogeneous and metabolically active biofilms on the surface of graphite-paste electrodes were obtained, which can be used as the basis of sensors.

From the point of view of use in biosensor devices, it is interesting to study the biocatalytic properties of microorganisms in biofilms. In this work, ferrocene was chosen as a mediator due to the fact that the electrochemical reaction with its participation does not depend on the pH of the medium, which makes it possible to use the optimal pH of the working electrolyte for the corresponding microorganism. In addition, the low solubility of ferrocene in water makes it possible to modify the graphite paste by immobilizing the mediator on the surface of the working electrode [33].

The method of cyclic voltammetry and Nicholson-Shine modeling for these systems (Equation (1)) [34] was used to determine the rate constant of the interaction of microorganisms with the electron transport mediator.

(1) $$\frac{I_{k}}{I_{d}}=\sqrt{\frac{k_{\mathrm{int}}\left[E\right]RT}{nFv}},$$

where *I_k_*—the limiting current in the presence of a substrate, μA; *I_d_*—the limiting current in the absence of a substrate, μA; *k* *_interaction_*—the rate constant of the interaction between the mediator and the biomaterial; [*E*]—cell titer, mg/L; *R*—the universal gas constant, J/(mol K); *T*—temperature, *K*; *n*—the number of transferred electrons; F—the Faraday constant, C/mol; *ν*—sweep speed, V/s.

**Figure 2:**
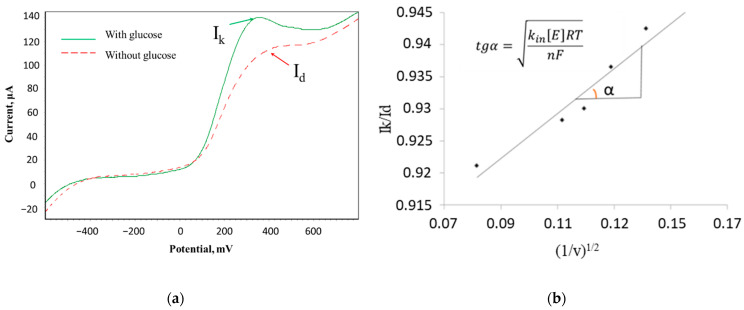
Determination of the mediator interaction constant with activated sludge bacteria in a biofilm by cyclic voltammetry. (**a**) Change in the strength of the anodic current in the presence and in the absence of a substrate for a biofilm of microorganisms; (**b**) dependence of the ratio of limiting currents in the presence and in the absence of a substrate on the reciprocal value of the parameter 1/*ν*^1/2^ in the “activated sludge biofilm–ferrocene mediator” system.

Dependences of the ratio of limiting anode currents in the presence and in the absence of glucose (*I*_k_/*I*_d_) on the value of (1/*ν*)^1/2^ were obtained to determine the rate constants; *k* interaction was found from the tangent of the slope of the linear regression. A typical view of the voltammograms and the calculated graph is shown in Figure 2.

The rate constant of the ferrocene mediator interaction with activated sludge microorganisms was 0.21 ± 0.02 dm^3^/(g s) when using its suspension and 0.38 ± 0.02 dm^3^/(g s) when using biofilm. Therefore, the biosensor based on activated sludge biofilm has the best characteristics, which makes it the most promising for further use. It should be noted that the rate constant of mediator interaction with microorganisms in bacterial biofilms is higher than in suspension. This fact can be associated with a much better contact of their enzyme systems with the electrode surface.

**Figure 3:**
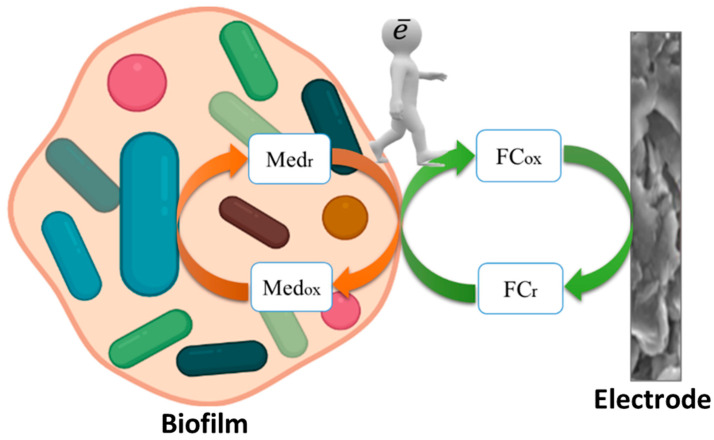
The mechanism of electron transfer in the system “graphite-paste electrode–ferrocene–endogenous electron transport mediators microorganisms biofilm”.

Since it is known that the matrix of biofilms can contain electroactive substances [15,24], such contact can be provided due to the emergence of a two-mediator system “graphite-paste electrode–ferrocene–endogenous mediator of the matrix microorganisms” (Figure 3). Thus, the exogenous mediator ferrocene can transfer electrons to the endogenous mediator in the polysaccharide matrix, and the endogenous mediator interacts more effectively with bacterial enzyme systems than ferrocene. Similar systems with two exogenous mediators previously showed high efficiency in bioelectrocatalysis [35,36].

### 3.2. Using Carbon Nanotubes to Create Electroactive Biofilms on the Electrodes Surface

It is known that microbial biofilms can be electroactive and can be used in biosensors and biofuel cells without the additional introduction of an exogenous electron transport mediator, performing electron transfer through extracellular polymers [19,37]. It is known from the literature that carbon nanotubes have a developed surface, high conductivity, and mechanical strength. The morphology of carbon nanotubes contributes to an increase in sensitivity, selectivity, and response stability compared with an unmodified sensor. They contribute to an increase in the surface area of the electrode, which increases the binding of microorganisms to the biofilm [38]. The biofilms that formed in this work did not exhibit electroactive properties when growing on the surface of graphite-paste electrodes. The surface of graphite-paste electrodes was modified with carbon nanotubes to create electrically conductive biofilms, after which electroactive properties were observed in the resulting biofilms. 

**Figure 4:**
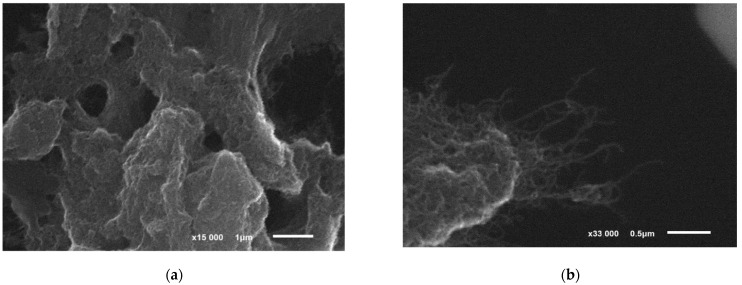

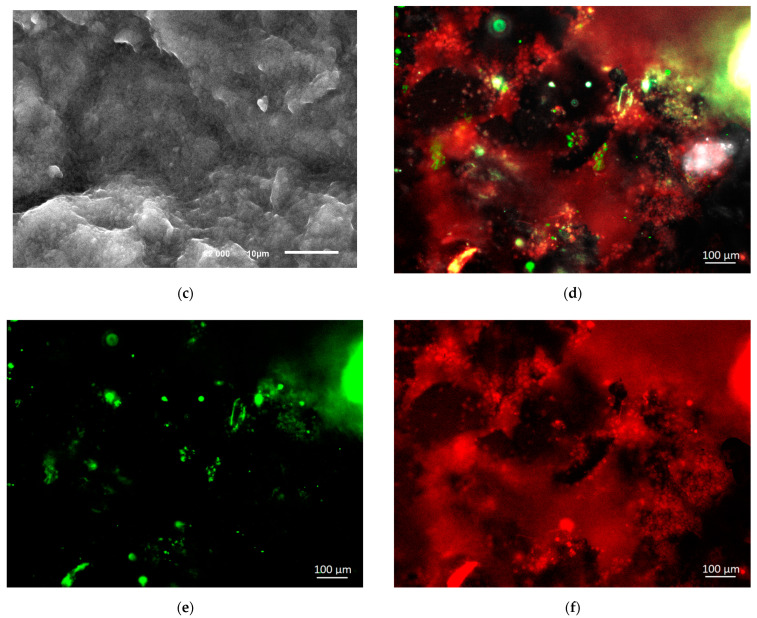
Microscopic images of the GPE surface: (**a**) SEM image after CNT modification; (**b**) SEM image after CNT modification (enlarged scale); (**c**) SEM image of the biofilm on the surface of the modified GPE; (**d**) integrated image of the surface of the nanostructured electrode, obtained by the LKSM method; (**e**) image of the surface of the nanostructured electrode in the conA dye display mode, obtained by the LKSM method; (**f**) image of the surface of the nanostructured electrode in the display mode of the SYTO 11 dye, obtained by the LKSM method.

The formation of biofilms of microorganisms on the surface of a modified graphite-paste electrode was shown using scanning electron microscopy and laser confocal microscopy (Figure 4).

When using carbon nanotubes, they are evenly distributed on the electrode surface, which can significantly facilitate the transfer of electrons from microorganisms in biofilms to the electrode (Figure 4a,b). In Figure 4b, CNT filaments on graphite particles are especially clearly visible, on which an electroactive biofilm can form. As can be seen from Figure 4c,d, the biofilm is successfully formed on the surface of the modified CNT electrode, and the density of microorganisms is higher than on the electrode without CNT modification (Figure 4e,f). The electrode surface is almost invisible under the biofilm layer, which is also explained by its high density.

**Figure 5:**
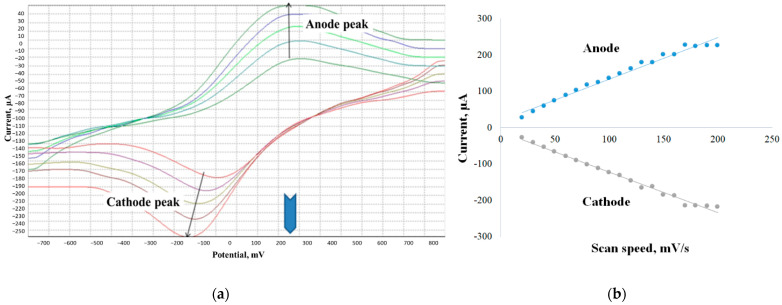
Study of electroactive biofilms by cyclic voltammetry: (**a**) cyclic voltammetric dependence for an electrode with a biofilm of activated sludge ferrocene and CNT, at different sweep speeds; (**b**) dependence of the current on the potential sweep rate when using an activated sludge biofilm and CNT.

The method of cyclic voltammetry (CV) was used to study the electrochemical features of the functioning of electroactive biofilms on the nanostructured surface of the electrode. Cyclic voltammograms are quite convenient for finding the limiting stages of electrochemical processes, since, in the case of slow electron diffusion to the electrode, the limiting current is directly proportional to the square root of the sweep rate. If the transfer of electrons from microorganisms is fast and the process is limited by the surface reaction on the electrode, the current is proportional to the sweep rate [39]. The identification of the limiting stage made it possible to apply the Nicholson model (2) [39] and the Laviron model [40] (3) to find the heterogeneous rate constant of electron transfer to the electrode (Figure 5).

(2) $$k_{S}=\psi \sqrt{\pi \frac{nFv}{RT}D},$$

(3) $$\log(k_{S})=\alpha \log(1-\alpha )+(1-\alpha )\log\alpha -\log(\frac{RT}{nFv})-\frac{\alpha (1-\alpha )nF\Delta E}{2.3RT},$$

where *k_s_*—the heterogeneous rate constant of the electrochemical system (s^−1^ cm); ψ—parameter affecting the potential difference of the peaks (Δ*E*, mV); *n*—the number of participating electrons; F—the Faraday number (C/mol); *ν*—the potential sweep rate (V/s); *R*—the universal gas constant (J mol/K); *T*—temperature (K); *D*—the diffusion coefficient (cm^2^/s); π is a mathematical constant; α—the transfer coefficient of the cathodic process; (1 − α)—anodic process transfer coefficient; Δ*E*—the potential difference between the anode and cathode peaks (V).

The obtained values of the heterogeneous rate constants of electron transfer to the electrode and the rate constants of the interaction of mediator compounds with microorganisms for the activated sludge biofilm with carbon nanotubes were compared with the values of the rate constants in the system with the exogenous mediator (ferrocene) (Table 1).

From the data presented in Table 1, it can be seen that the rate constant of the interaction of the polymeric mediator compound with microorganisms in biofilms was significantly higher than when using the ferrocene mediator. The results obtained confirm the proposed model of electron transfer in biofilms due to the presence of redox-active particles in the polysaccharide matrix. Such endogenous mediator substances obviously interact much more effectively with the enzyme systems of microorganisms, but they are characterized by a somewhat lower rate of interaction with the electrode compared with ferrocene, but higher than many other exogenous mediators [33].

**Figure 6:**
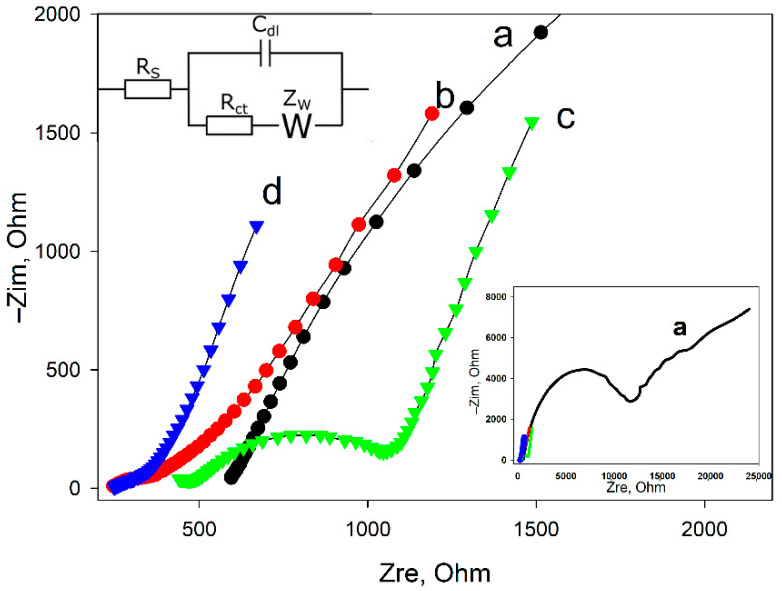
Nyquist diagrams for pure GPE (a), GPE/CNT (b), GPE/activated sludge (c), and GPE/CNT/activated sludge (d) containing 5 mm Fe(CN)_6_^3−^/Fe(CN)_6_^4−^. Insets: full Nyquist diagram for GPE (a); Randles modified equivalent circuit used to obtain electrode parameters.

Electrochemical impedance spectroscopy was used for an in-depth study of the process of electrode modification with an electroactive biofilm. Figure 6 shows the impedance spectra in the form of Nyquist diagrams for four variants of electrode modifications: unmodified GPE, GPE/CNT, GPE/activated sludge biofilm, and GPE/CNT/activated sludge biofilm. The resulting spectra were processed using a modified standard Randle equivalent circuit (inset in Figure 6), which consists of charge transfer resistance (R_ct_), solution ohmic resistance (R_s_), Warburg element (Z_w_), and electric double layer capacitance (C_dl_). The diameter of the semicircle in the high-frequency region reflects the value of R_ct_, and the curve of the diagram in the low-frequency region indicates the presence of diffusion restrictions, expressed by the Warburg element. The R_ct_ value for pure GPE was 11,090 Ohm, while with CNT modification it significantly decreased to 50 Ohm, indicating an increased conductivity on the electrode surface, which can be explained by the excellent conductive properties of CNT. R_ct_ decreased to 437 Ohm when modifying a graphite paste electrode with an activated sludge suspension, and R_ct_ was 197 Ohm for a completely prepared HPE/CNT/activated sludge composition. Thus, it is worth noting that the use of CNT made it possible to achieve a significant decrease in the total resistance of the system, even taking into account the small insulating effect of immobilized cells on the electrode conductivity, which was confirmed using the method of cyclic voltammetry.

It was observed that activated sludge cells without the use of carbon nanotubes also significantly reduced the charge transfer resistance of the electrode. These results confirm the release of extracellular redox-active compounds by the biofilm of activated sludge; however, their contact with the electrode surface is more difficult without the presence of CNT in the system.

**Figure 7:**
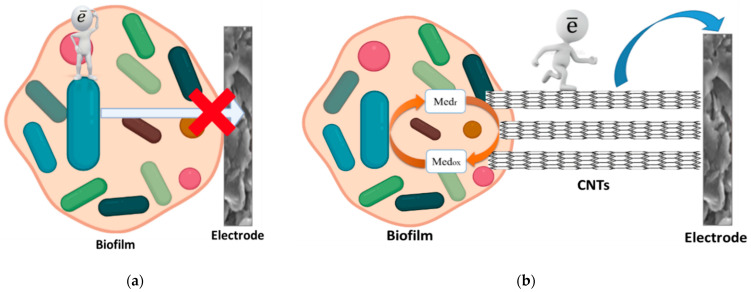
Schematic diagram of the operation of receptor systems based on activated sludge biofilms: (**a**) without creation of a nanostructured surface; (**b**) after the creation of a nanostructured surface due to the electrode modification with CNT.

Therefore, it can be assumed that the creation of the nanostructured surface of the graphite paste electrode described in the work leads to the incorporation of carbon nanotubes into the polymer matrix of the biofilm (Figure 7). The transfer of electrons from the biofilm to the electrode surface without CNT can be hindered at the interface. The inclusion of CNT in a biofilm removes the restrictions that have arisen and makes it possible to transfer electrons to the electrode directly from the extracellular matrix. Another advantage of modifying the CNT electrode is to increase the effective surface area of the electrode and increase the sensitivity of the analysis.

### 3.3. Metrological and Analytical Characteristics of the Developed Bod Biosensors

**Figure 8:**
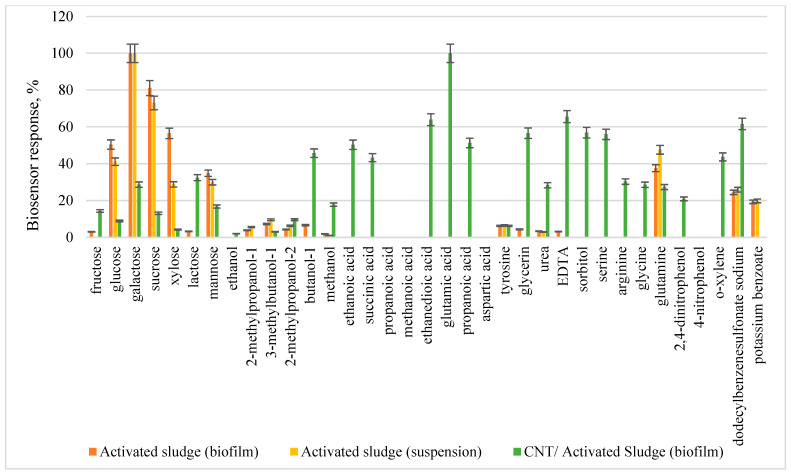
Comparative diagram of the substrate specificity of receptor elements based on biofilms and activated sludge suspension.

The developed biosensor should register the biochemical oxidation of a wide range of organic substrates for BOD_5_ analysis; therefore, the substrate specificity of biosensors based on the developed systems was studied (Figure 8).

The analysis of substrate specificity allowed us to conclude that the profile of substrate oxidation changes upon transition from a suspension of microorganisms to an electroactive biofilm. In general, a biosensor based on an activated sludge biofilm and CNT makes it possible to register the oxidation of the widest range of organic compounds, which ensures the correctness of the BOD_5_ analysis results. An increase in the amount of oxidizable substrates when using an electroactive biofilm is associated with a more efficient interaction of extracellular redox-active polymeric substances with enzyme systems of microorganisms compared with ferrocene.

**Figure 9:**
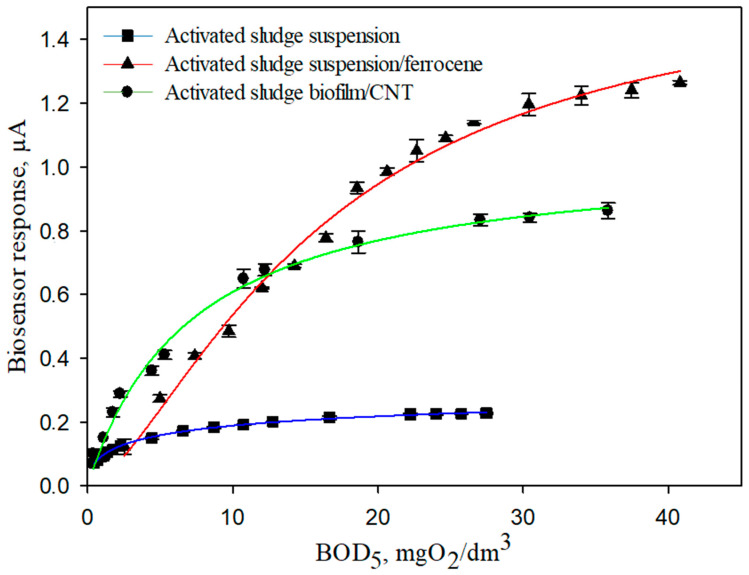
Calibration dependences of the sensor response on the concentration of BOD_5_ for the created biosensor systems.

Hyperbolic dependences of the sensor response on BOD_5_ were obtained (Figure 9) to quantify the content of analytes in the sample. The answers were approximated by the Michaelis–Menten equation:

(4) $$V=\frac{V_{\max}{\left[S\right]}^{}}{K_{M}{}^{}+[S]},$$

where V—the rate of the enzymatic reaction; V_max_—the sensitivity to the enzymatic reaction; K_M_—the apparent Michaelis constant; [S]—the initial concentration of the substrate.

Characteristics such as long-term and operational stability were investigated for stability analysis and perceptual stability in long-term analysis. Glucose-glutamate mixture (GGM) was chosen as a substrate, which was considered as a standard in international practice with the taste of BOD_5_ [3]. Table 2 presents all the characteristics of the developed mediator biosensors based on the use and literature products.

According to the obtained data, it can be noted that the lower limit of the determined BOD_5_ concentrations of the developed biosensors using gels based on ferrocene and CNT exceeded many known analogues. CNT makes it possible to increase the efficiency of electron transfer by increasing the constant of heterogeneous electron transfer to the electrode during bioelectrocatalysis in an electroactive biofilm. Based on the obtained results, a biosensor based on an electroactive biofilm of activated sludge is promising in terms of selectivity, long-term stability, and the range of detectable concentrations for creating a BOD biosensor.

The developed BOD biosensor based on an electroactive biofilm makes it possible to obtain a stable signal, since the relative standard deviation did not exceed 6%, and the time of stable operation was more than 50 days. It should be noted that this system allows the analysis of natural waters, where the BOD_5_ value can be quite low and amount to ~1 mg/dm^3^ (pure natural waters). The developed biosensor based on an activated sludge biofilm with CNT is not inferior to the already known analogs of biosensors based on activated sludge, and even surpasses them in many characteristics [11,15,24,41]. It is the most sensitive and takes less time for a single analysis, making it promising for BOD_5_ analysis in surface water and wastewater.

### 3.4. The Influence of Negative Environmental Factors on the Developed Receptor System

**Figure 10:**
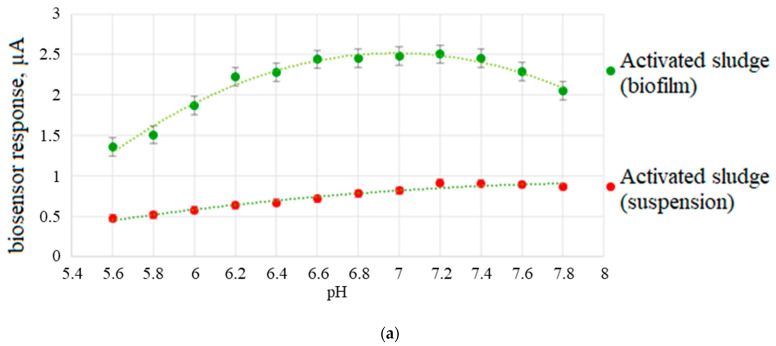

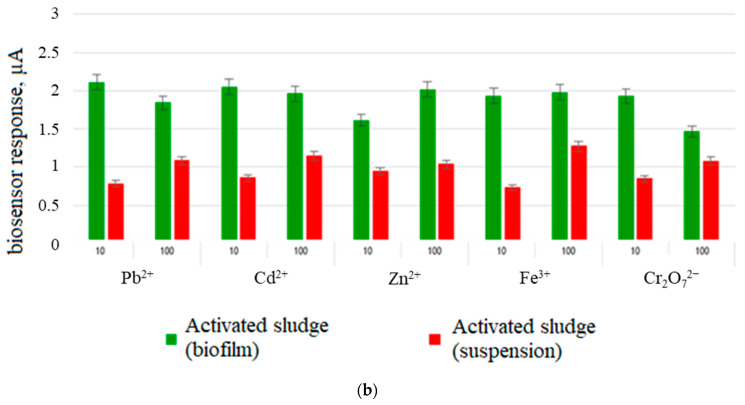
Dependence of the response of a biosensor based on a suspension of microorganisms and an activated sludge biofilm on the pH of the medium (**a**) and the presence of heavy metal ions (**b**).

Wastewater from industrial enterprises can contain organic compounds oxidized by microorganisms in the composition of the bioreceptor, and also contain inorganic substances that have a negative effect on the oxidative activity of microorganisms. Therefore, an important factor in the development of a biosensor for determining BOD is the study of the influence of negative environmental factors on the developed receptor system. The pH of the medium is one of the factors affecting the activity of cellular enzymes and the sensitivity of the bioreceptor to various substrates. In this work, the change in the oxidative activity of activated sludge bacterial cells in the form of biofilms and free-living microorganisms was studied with varying pH from 5.6 to 7.8 (the lower and upper limits of the possible pH of the KH_2_PO_4_/Na_2_HPO_4_ buffer system). Figure 10a shows the dependence of the response of a biosensor based on an activated sludge biofilm on the pH of the medium.

It was shown that the maximum responses of biosensors based on biofilm of activated sludge cells were observed in the pH range of 6.6–7.2, which corresponds to the literature data [42]. It should be noted that microorganisms in biofilms exhibit greater physiological activity in this pH range than microorganisms in suspension. Based on revenue data developed using a biofilm-based biosensor, the optimum concentration for analyzed samples is pH 6.8 to 7.0.

The main factor that can reduce the response of the biosensor and even lead to the death of the bioreceptor is the presence of heavy metal ions in wastewater. The mechanisms of their interaction with enzymes are different, for example, the substitution of physiologically important cations and non-metal-containing oxyanions in the active centers of enzymes, the binding of functional sulfidehydryl groups, etc. [43]. 

The dependence of the oxidative capacity of the used microorganisms in the form of biofilms and suspensions on the presence of Cd^2+^, Pb^2+^, Zn^2+^, Fe^3+^, and Cr_2_O_7_^2−^ ions in the solution in the range of concentrations exceeding the MAC for water bodies by 10 and 100 times was studied to study the inhibitory effect of heavy metal compounds [44]. The measured parameter was the response of the biosensor to the addition of glucose glutamate mixture in the presence of salts. Figure 10b shows a comparison of the effect of heavy metals on the biofilm and the activated sludge bacterial suspension. When the maximum permissible concentrations was exceeded by 100 times for all heavy metal ions under study, the decrease in the responses of the biofilm-based receptor system was not more than 20%, which may indicate the protective effect of biofilms formed on the graphite electrode surface. Thus, the use of microorganisms’ biofilms in receptor systems can be a promising approach to improve the stability of biosensor systems.

### 3.5. Approbation of the Developed Biosensor

**Figure 11:**
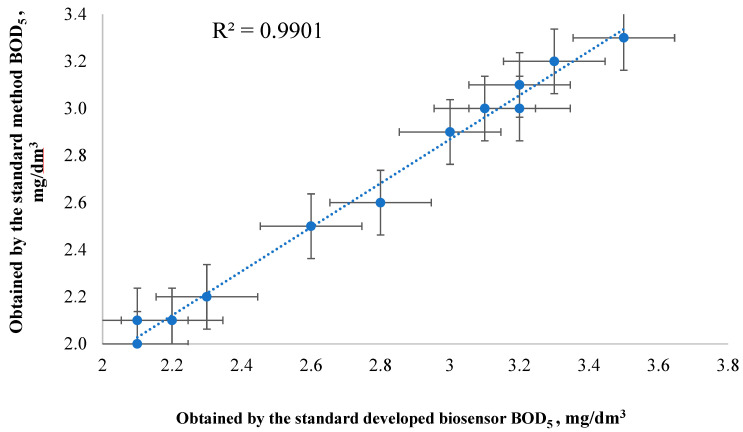
Linear dependence of the results of BOD_5_ analysis by the standard method and using the developed biosensor.

Approbation of a biosensor based on a biofilm of activated sludge with CNT was carried out on twelve samples of natural and waste water. Sampling and determination of BOD_5_ by the standard method was carried out in accordance with the current regulatory documents (Figure 11).

Statistical processing (Modified Student’s test) of the obtained results showed that the data obtained by both methods were not significantly different. The developed biosensor can be effectively used as an alternative to the standard BOD_5_ analysis.

## 4. Conclusions

Therefore, electroactive biofilms of activated sludge grown on the surface of a graphite-paste electrode modified with carbon nanotubes were formed in this work. A complex of microscopy methods confirmed the formation of biofilms: optical microscopy with phase contrast, scanning electron microscopy, and laser confocal microscopy.

The rate constants of the interaction of microorganisms with the mediator and the heterogeneous rate constant of electron transfer were determined using the method of cyclic voltammetry. Based on the obtained kinetic constants and electrochemical impedance spectroscopy data, it was shown that the modification of the CNT electrode surface makes it possible to create electroactive biofilms in which electron transfer occurs with the participation of extracellular redox-active polymers and nanotubes integrated into the matrix.

An analysis of the metrological and analytical characteristics of the created biosensors showed that the high sensitivity of the BOD biosensor based on the electroactive biofilm of activated sludge makes it possible to analyze almost any surface water samples. The results of the analysis of natural water samples obtained by the biosensor and the standard method differed insignificantly, which indicates the prospect of using the developed biosensor as a prototype for the development of serial BOD analyzers.

## Figures and Tables

**Table 1:** Rate constants of mediator interaction with microorganisms and rate constants of heterogeneous electron transfer to a graphite-paste electrode.

System	Rate Constant of Mediator Interaction with Microorganisms, dm^3^/(g s)	Constants of Heterogeneous Electron Transfer, cm s^−1^
CNT/activated sludge biofilm	0.79 ± 0.03	0.34 ± 0.02
CNT/activated sludge suspension	-	-
Ferrocene/activated sludge biofilm	0.38 ± 0.02	0.4 ± 0.1
Ferrocene/activated sludge suspension	0.21 ± 0.02	0.4 ± 0.1

**Table 2:** Comparative characteristics of analytical and metrological characteristics of mediator biosensors.

Biomaterial/Conduction System	Long-Term Stability, Days	Relative Standard Deviation, % (*n* = 15, *p* = 0.95)	Linear Bod_5_ Range, mg/dm^3^	Analysis Time, min	Detection Limit, mgO_2_/dm^3^	Reference
Activated sludge suspension/ferrocene	29	5.12	1.31–1.6	5	0.44	This study
Activated sludge biofilm/ferrocene	52	6.79	0.87–31	5	0.29	This study
CNT/Electroactive Activated Sludge Biofilm	53	5.96	0.41–23	5	0.14	This study
Biofilm based on a community of microorganisms	-	-	20–500	-	-	[41]
Activated sludge/methylene blue/graphene	65	2.9	1–100	9	0.33	[11]
Biofilm/hydrogel based on reduced graphene oxide	60	-	2–64	10	0.4	[15]
Bacteria from activated sludge/covalently bound ferrocene/CNT	50	5.0	0.1–4.5	5	-	[24]

## Data Availability

Not applicable.

## References

[1] Hu J., Li Y., Gao G., Xia S. (2017). A Mediated BOD Biosensor Based on Immobilized B. Subtilis on Three-Dimensional Porous Graphene-Polypyrrole Composite. Sensors.

[2] Yu D., Yong Y.-C., Liu C., Fang Y., Bai L., Dong S. (2017). New applications of genetically modified Pseudomonas aeruginosa for toxicity detection in water. Chemosphere.

[3] (2019). ISO Water Quality—Determination of Biochemical Oxygen Demand after n Days (BODn), Part 1: Dilution and Seeding Method with Allylthiourea Addition.

[4] Jouanneau S., Recoules L., Durand M.J., Boukabache A., Picot V., Primault Y., Lakel A., Sengelin M., Barillon B., Thouand G. (2014). Methods for assessing biochemical oxygen demand (BOD): A review. Water Res..

[5] Gupta N., Renugopalakrishnan V., Liepmann D., Paulmurugan R., Malhotra B.D. (2019). Cell-based biosensors: Recent trends, challenges and future perspectives. Biosens. Bioelectron..

[6] Ejeian F., Etedali P., Mansouri-Tehrani H.-A., Soozanipour A., Low Z.-X., Asadnia M., Taheri-Kafrani A., Razmjou A. (2018). Biosensors for wastewater monitoring: A review. Biosens. Bioelectron..

[7] Singh S., Singh J., Singh H. (2021). Chapter 3-Chemical oxygen demand and biochemical oxygen demand. Green Sustainable Process for Chemical and Environmental Engineering and Science.

[8] Chadha U., Bhardwaj P., Agarwal R., Rawat P., Agarwal R., Gupta I., Panjwani M., Singh S., Ahuja C., Selvaraj S.K. (2022). Recent progress and growth in biosensors technology: A critical review. J. Ind. Eng. Chem..

[9] Zhao L., He L., Chen S., Zou L., Zhou K., Ao X., Liu S., Hu X., Han G. (2017). Microbial BOD sensors based on Zr (IV)-loaded collagen fiber. Enzyme Microb. Technol..

[10] Yudina N.Y., Arlyapov V.A., Chepurnova M.A., Alferov S.V., Reshetilov A.N. (2015). A yeast co-culture-based biosensor for determination of waste water contamination levels. Enzyme Microb. Technol..

[11] Niyomdecha S., Limbut W., Numnuam A., Asawatreratanakul P., Kanatharana P., Thavarungkul P. (2017). A novel BOD biosensor based on entrapped activated sludge in a porous chitosan-albumin cryogel incorporated with graphene and methylene blue. Sens. Actuators B Chem..

[12] Vassilev I., Dessì P., Puig S., Kokko M. (2022). Cathodic biofilms–A prerequisite for microbial electrosynthesis. Bioresour. Technol..

[13] Commault A.S., Lear G., Bouvier S., Feiler L., Karacs J., Weld R.J. (2016). Geobacter-dominated biofilms used as amperometric BOD sensors. Biochem. Eng. J..

[14] Yi Y., Zhao T., Xie B., Zang Y., Liu H. (2020). Dual detection of biochemical oxygen demand and nitrate in water based on bidirectional Shewanella loihica electron transfer. Bioresour. Technol..

[15] Liu L., Zhai J., Zhu C., Gao Y., Wang Y., Han Y., Dong S. (2015). One-pot synthesis of 3-dimensional reduced graphene oxide-based hydrogel as support for microbe immobilization and BOD biosensor preparation. Biosens. Bioelectron..

[16] Lu S., Tian J., Sun W., Meng J., Wang X., Fu X., Wang A., Lai D., Liu Y., Zhou L. (2014). Bis-naphtho-γ-pyrones from fungi and their bioactivities. Molecules.

[17] Busalmen J.P., Esteve-Núñez A., Berná A., Feliu J.M. (2008). C-type cytochromes wire electricity-producing bacteria to electrodes. Angew. Chem. Int. Ed..

[18] Light S.H., Su L., Rivera-Lugo R., Cornejo J.A., Louie A., Iavarone A.T., Ajo-Franklin C.M., Portnoy D.A. (2018). A flavin-based extracellular electron transfer mechanism in diverse Gram-positive bacteria. Nature.

[19] Ter Heijne A., Pereira M.A., Pereira J., Sleutels T. (2021). Electron Storage in Electroactive Biofilms. Trends Biotechnol..

[20] Molenaar S.D., Sleutels T., Pereira J., Iorio M., Borsje C., Zamudio J.A., Fabregat-Santiago F., Buisman C.J.N., Ter Heijne A. (2018). In situ biofilm quantification in bioelectrochemical systems by using optical coherence tomography. ChemSusChem.

[21] Melnikov P.V., Alexandrovskaya A.Y., Naumova A.O., Popova N.M., Spitsyn B.V., Zaitsev N.K., Yashtulov N.A. (2021). Modified Nanodiamonds as a Means of Polymer Surface Functionalization. From Fouling Suppression to Biosensor Design. Nanomaterials.

[22] Carmona-Martinez A.A., Harnisch F., Fitzgerald L.A., Biffinger J.C., Ringeisen B.R., Schröder U. (2011). Cyclic voltammetric analysis of the electron transfer of Shewanella oneidensis MR-1 and nanofilament and cytochrome knock-out mutants. Bioelectrochemistry.

[23] Mashkour M., Rahimnejad M., Raouf F., Navidjouy N. (2021). A review on the application of nanomaterials in improving microbial fuel cells. Biofuel Res. J..

[24] Qi X., Wang S., Li T., Wang X., Jiang Y., Zhou Y., Zhou X., Huang X., Liang P. (2021). An electroactive biofilm-based biosensor for water safety: Pollutants detection and early-warning. Biosens. Bioelectron..

[25] Arlyapov V.A., Kharkova A.S., Kurbanaliyeva S.K., Kuznetsova L.S., Machulin A.V., Tarasov S.E., Melnikov P.V., Ponamoreva O.N., Alferov V.A., Reshetilov A.N. (2021). Use of biocompatible redox-active polymers based on carbon nanotubes and modified organic matrices for development of a highly sensitive BOD biosensor. Enzyme Microb. Technol..

[26] Luong J.H.T., Glennon J.D., Gedanken A., Vashist S.K. (2017). Achievement and assessment of direct electron transfer of glucose oxidase in electrochemical biosensing using carbon nanotubes, graphene, and their nanocomposites. Microchim. Acta.

[27] Feizabadi M., Ajloo D., Soleymanpour A., Faridnouri H. (2018). Study of electron transport in the functionalized nanotubes and their impact on the electron transfer in the active site of horseradish peroxidase. J. Phys. Chem. Solids.

[28] Burton E., Yakandawala N., LoVetri K., Madhyastha M.S. (2007). A microplate spectrofluorometric assay for bacterial biofilms. J. Ind. Microbiol. Biotechnol..

[29] Aleksandrovskaya A.Y., Melnikov P.V., Safonov A.V., Abaturova N.A., Spitsyn B.V., Naumova A.O., Zaitsev N.K. (2019). The Effect of Modified Nanodiamonds on the Wettability of the Surface of an Optical Oxygen Sensor and Biological Fouling During Long-Term in Situ Measurements. Nanotechnol. Russ..

[30] Kharkov A.S., Arlyapov V.A., Turovskaya A.D., Avtukh A.N., Starodumova I.P., Reshetilov A.N. (2019). Mediator BOD-biosensor based on microbial cells isolated from activated sludge. Appl. Biochem. Microbiol..

[31] Jordan M.A., Welsh D.T., John R., Catterall K., Teasdale P.R. (2013). A sensitive ferricyanide-mediated biochemical oxygen demand assay for analysis of wastewater treatment plant influents and treated effluents. Water Res..

[32] Castro L., Blázquez M.L., González F., Muñoz J.A., Ballester A. (2019). Anaerobic bioreduction of Jarosites and Biofilm formation by a natural microbial consortium. Minerals.

[33] Kharkova A.S., Arlyapov V.A., Turovskaya A.D., Shvets V.I., Reshetilov A.N. (2020). A mediator microbial biosensor for assaying general toxicity. Enzyme Microb. Technol..

[34] Nicholson R.S., Shain I. (1964). Theory of stationary electrode polarography. Single scan and cyclic methods applied to reversible, irreversible, and kinetic systems. Anal. Chem..

[35] Kharkova A.S., Arlyapov V.A., Ilyukhina A.S., Ponamoreva O.N., Alferov V.A., Reshetilov A.N. (2021). A kinetic approach to the formation of two-mediator systems for developing microbial biosensors as exemplified by a rapid biochemical oxygen demand assay. 3 Biotech.

[36] Zaitseva A.S., Arlyapov V.A., Yudina N.Y., Alferov S.V., Reshetilov A.N. (2017). Use of one-and two-mediator systems for developing a BOD biosensor based on the yeast Debaryomyces hansenii. Enzyme Microb. Technol..

[37] Zhuang Z., Yang G., Zhuang L. (2022). Exopolysaccharides matrix affects the process of extracellular electron transfer in electroactive biofilm. Sci. Total Environ..

[38] Jariwala D., Sangwan V.K., Lauhon L.J., Marks T.J., Hersam M.C. (2013). Carbon nanomaterials for electronics, optoelectronics, photovoltaics, and sensing. Chem. Soc. Rev..

[39] Nicholson R.S. (1965). Theory and application of cyclic voltammetry for measurement of electrode reaction kinetics. Anal. Chem..

[40] Laviron E.J.J. (1979). General expression of the linear potential sweep voltammogram in the case of diffusionless electrochemical systems. J. Electroanal. Chem. Interfacial Electrochem..

[41] Guo F., Liu Y., Liu H. (2021). Hibernations of electroactive bacteria provide insights into the flexible and robust BOD detection using microbial fuel cell-based biosensors. Sci. Total Environ..

[42] Wu S.-L., Wei W., Wang Y., Song L., Ni B.-J. (2022). Transforming waste activated sludge into medium chain fatty acids in continuous two-stage anaerobic fermentation: Demonstration at different pH levels. Chemosphere.

[43] Berks B.C., Ferguson S.J., Moir J.W.B., Richardson D.J. (1995). Enzymes and associated electron transport systems that catalyse the respiratory reduction of nitrogen oxides and oxyanions. Biochim. Biophys. Acta Bioenerg..

[44] (2003). Maximum Permissible Concentrations (Mpc) of Chemicals in the Water of Water Bodies of Household Drinking and Cultural Water Use: Hygienic Standards.

